# Study on the Use of Microbial Cellulose as a Biocarrier for 1,3-Dihydroxy-2-Propanone and Its Potential Application in Industry

**DOI:** 10.3390/polym10040438

**Published:** 2018-04-14

**Authors:** Lidia Stasiak-Różańska, Justyna Płoska

**Affiliations:** 1 Department of Biotechnology, Microbiology and Food Evaluation, Faculty of Food Sciences, Warsaw University of Life Sciences-SGGW, Nowoursynowska St. 166, 02-787 Warsaw, Poland; 2 Faculty of Horticulture, Biotechnology and Landscape Architecture, Warsaw University of Life Sciences-SGGW, Nowoursynowska St. 166, 02-787 Warsaw, Poland; justyna.ploska@02.pl

**Keywords:** microbial cellulose, bacterial cellulose, dihydroxyacetone, biomaterial, biocarrier, vitiligo

## Abstract

Can microbial cellulose (MC) be used as a bio-carrier for 1,3-dihydroxy-2-propanone (DHA)? The aim of this study was to examine the possibility of using MC as a biomaterial for DHA transferring into the stratum corneum and inducing changes in skin color. The MC patches were obtained from *Gluconacetobacter xylinus* strain and incubated in solutions with various concentrations of DHA (g·L^−1^: 20; 50; 80; 110) at 22 °C for 24 h. Afterwards; the patches were applied onto the skin for 15, 30, or 60 min. Skin color changes were assessed visually compared to a control patches without DHA. The intensity of skin color was increasing with the increase of DHA concentration and time of patches application. Application of MC patches with DHA (50 g·L^−1^) for 30 min ensured the color which was considered the closest to the desired natural tan effect. MC patches containing DHA can be biocarriers enabling DHA transport into the stratum corneum and causing skin color changes. Study results indicate a new possibility for industrial applications of MC; e.g., as a biocarrier in masking the symptoms of vitiligo or production of self-tanning agents in the form of masks.

## 1. Introduction

Acetic acid bacteria (AAB) are Gram-negative, aerobic rods [1]. The best known feature of these microorganisms is their capability to oxidize ethanol to acetic acid. AAB can also produce many other compounds, such as gluconic acid, that are currently widely used in the industry. Gluconic acid is used in the food industry for pickling food and as a pH stabilizer (in the form of an ester) in powdered mixtures for baking cakes and bread and an additive to sausages and other meat products [2]. AAB are also a source of a valuable exopolysaccharide—levan, which can be used as probiotic fiber [3,4]. In recent years, many scientific articles have appeared about industrial applications of other important metabolites of AAB—microbial cellulose and dihydroxyacetone [5,6].

**Figure 1:**
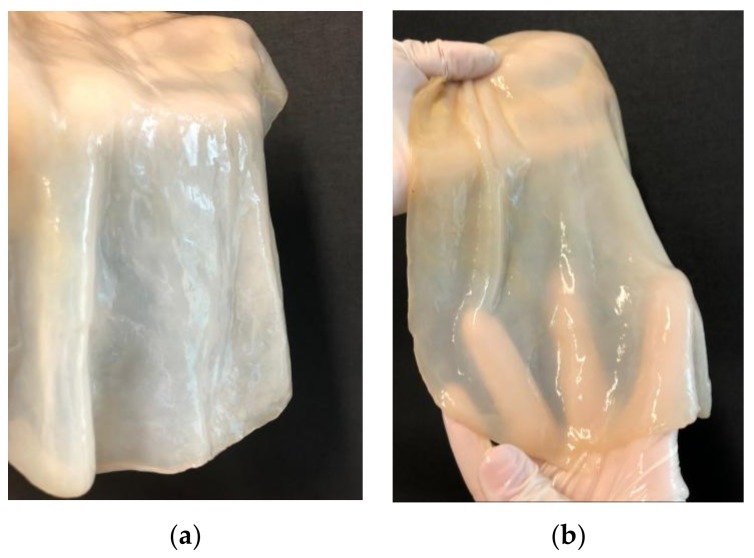
Microbial cellulose obtained from *Gluconacetobacter xylinus* strain, before removing bacterial cells (**a**), after removing bacterial cells (**b**).

Microbial cellulose (MC, synonym: bacterial cellulose) is a natural polymer, produced by some species of AAB, including *Gluconacetobacter xylinus* (synonymous *Acetobacter xylinus*), Figure 1.

MC is composed of three-dimensional nano-fibrous networks of a linear polysaccharide polymer linked by-(1,4) glycosidic linkages. The thickness of MC nanofibres (in cross-section) is 5–10 × 30–35 nm [7]. This polymer has good mechanical properties, a high level of crystallinity, high water holding capacity, and excellent biocompatibility with human skin. These characteristics can be advantageous for regeneration of body organs, such as skin, bones, cartilage, nerves, heart, blood vessels, and dental implants [8]. MC is also characterized by high thermal stability. Rheological analyses showed its good consistency and viscosity. MC is a potential food additive and could also be used as a food packaging material. Its high textural stability during freeze–thaw cycles makes this polymer an effective additive to frozen food products [9]. MC shows some swelling properties which can depend on external factors (e.g., temperature, pH, ionic strength). Thanks to MC ability to swell, the MC-based hydrogels are developed as active delivery systems for drugs, proteins, and hormones [10]. 

MC is widely used both in the food industry (filler, food additive that reduces energy value, food type Nata de coco) as well as in cosmetics (stabilizer of creams, tonics, artificial nail component) [11]. In medicine, MC is the main component of bio-dressings. Their great advantage is the considerable flexibility and ease of penetration of water, oxygen, and solutions such as glucose, sucrose, NaCl, and KCl, which promote the process of wound healing. A positive effect of using cellulose dressings on pain relief, increased absorption of wound leaks and reductions in treatment costs and time has already been observed. Significant porosity of cellulose patches facilitates effective impregnation with antibiotics and other medicines, which enables their penetration to the wound treated with a biocellulose dressing. Such treatments minimize bacterial or fungal infections at the same time [12,13]. MC is called the “biomaterial of the future” and its application possibilities are still extended [14,15].

Microbial cellulose is a very stable material but when it is out of use it is degraded without leaving environmentally harmful footprints, thus forming a well-working circular economy. MC is an environmentally-friendly polymer [16].

Dihydroxyacetone (1,3-dihydroxy-2-propanone, DHA) belongs to ketotrioses and is a reducing sugar. In the crystalline state, it is a white substance with a refreshingly sweet taste and a characteristic smell [17]. DHA is classified on the GRAS list, which means it is safe for humans (www.fda.gov). It is a widely used compound in the food industry as a fragrance enhancer for thermally-processed products [18], and in pharmacy as an intermediate for the production of anticancer drugs [19]. DHA is also used in the production of polyethylene glycol, which is a component of antiperspirants, toothpastes, and hand hygiene products [20]. Furthermore, it is used on a large scale in the cosmetic industry as an active ingredient of all types of self-tanning products. Dihydroxyacetone is commonly found in sunless self-tanning lotions, creams, and sprays and it is delivered onto the skin by massaging and rubbing/patting. It is the only FDA approved color additive for use as a tanning agent. It binds in the stratum corneum and forms brown-black compounds called melanoids, giving the effect of a natural tan. DHA induces the formation of brown color complexes through glycosylation of amines or amino groups in skin proteins. The process is known as the Maillard reaction and involves the formation of free radicals. Pigment loss occurs through exfoliation of the stratum corneum. The main disadvantages of DHA delivery in form of self-tanning creams is the unpleasant smell after application which disappears with the brown-color skin effect. In some situations, the application of self-tanning cosmetics with DHA is impossible because it requires washing hands after each use of these products. Moreover, the application of DHA in cream formula cannot ensure an even delivery of the active ingredient to the skin surface. The final effect of skin coloring can be observed after about 10 h and usually it is too late to provide necessary correction of skin pigmentation [21,22,23]. The DHA property of skin coloring can be effectively used to relieve skin vitiligo [24,25].

**Figure 2:**
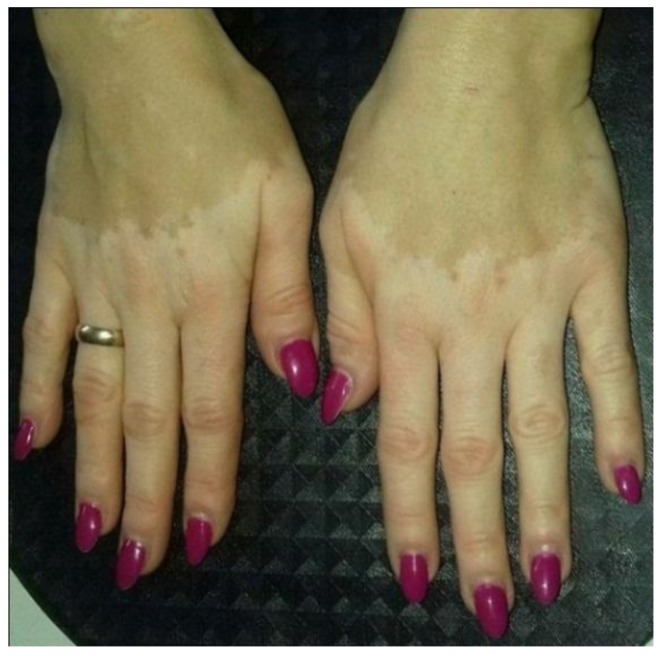
Hands of the patient with vitiligo.

Vitiligo is an autoimmune cutaneous disorder targeting melanocytes, manifesting itself as irregular multiple depigmented stains on the skin, especially on hands, Figure 2 [26].

No high-level standard methods for assessing vitiligo have been reported so far. This disease, at any stage, may diminish the comfort and quality of patient life [27]. Vitiligo is also characterized by destruction of hair follicles [28]. It currently afflicts 0.5–4.0% of the global population [25]. First line treatment includes topical corticosteroids, calcineurin inhibitors, and narrowband ultraviolet-B phototherapy [29]. There are many surgical methods for vitiligo treatment that have been used for over 30 years. Suction blister epidermal grafting (SBEG) is considered one of the simplest and most effective of them [30]. There are some modifications of this method such as SBEG with CO_2_ laser ablation which offers simple and safe treatment of vitiligo, especially for patients with small and stable vitiliginous lesions [31]. Patients with vitiligo can use self-tanning creams to partially cover the lighter stains on their skin [32]. Despite the well-known methods, there is still lack of inexpensive, popular and easily-accessible ways to treat of vitiligo symptoms.

The aim of this study was to examine the possibility of using microbial cellulose soaked in a DHA solution as a biocarrier, which ensures the bronze-color effect after its application on skin.

## 2. Materials and Methods

To produce MC the AAB strain *Gluconacetobacter xylinus* was used. The strain was obtain from the Colection of Pure Culture, Department of Biotechnology and Food Microbiology, Warsaw University of Life Sciences (Warsaw, Poland).

Growing medium (g·L^−1^): yeast extract 30, ethyl alcohol 20, pH 5.0.

Culture medium for MC production (g·L^−1^): yeast extract 30, glucose 30, ethyl alcohol 20, pH 5.0.

DHA (Merck, Hohenbrunn, Germany, Cat. No: 8.20482.0100) solutions in distilled water (g·L^−1^): 20, 50, 80 and 110, pH 4.8.

### 2.1. Growing culture of G. xylinus

50 cm^3^ of the growing medium was inoculated with pure culture of *G. xylinus*, incubation was carried out at 28 °C for 24 h on a reciprocating shaker, 200 rpm.

### 2.2. Production of MC by G. xylinus Strain

The MC medium (50 cm^3^) was inoculated with *G. xylinus*, incubated at 28 °C for 24 h on a reciprocating shaker, 200 rpm. Then, 20 cm^3^ of culture was transferred to 100 cm^3^ sterile flasks and the culture was grown stationary for 96 h at 28 °C. After this time, MC was collected from the surface of the medium. The obtained MC patches had a diameter of 55 mm and a thickness of 3 mm.

### 2.3. Soaking the MC with DHA

The MC patches were rinsed five times with sterile distilled water to remove *G. xylinus* cells, then neutralized by 0.1 M NaOH. Removal of AAB cells was checked microscopically.

The washed, neutralized, and cell-free MC patches were incubated in DHA solutions at 22 °C for 24 h. In addition, a control sample was prepared by incubating MC in water without DHA addition. 

### 2.4. Application MC with DHA on Skin

Three volunteers (without vitiligo) took a part in the application part of experiment. The patches were gently dried from DHA solutions with a sterile tissue, and then applied onto clean, dry skin on the front of the thigh. The application times were 15, 30, and 60 min.

All experiments were provided in three independent repeats.

All materials used in this study are safe for humans, classified as GRAS, and non-toxic. MC and DHA are commercially used compounds approved for contact with human skin.

### 2.5. Visual Assessment of Skin Browning Effects

The grade of skin color darkening was determined subjectively in this study by 15 independent observers 12 h after MC patches removal. In the assessment of skin color, a scale from 0 to 5 was adopted in which ‘0’ meant no change in skin color (control), and ‘5’ indicated the most intensive color obtained in the study.

**Figure 3:**
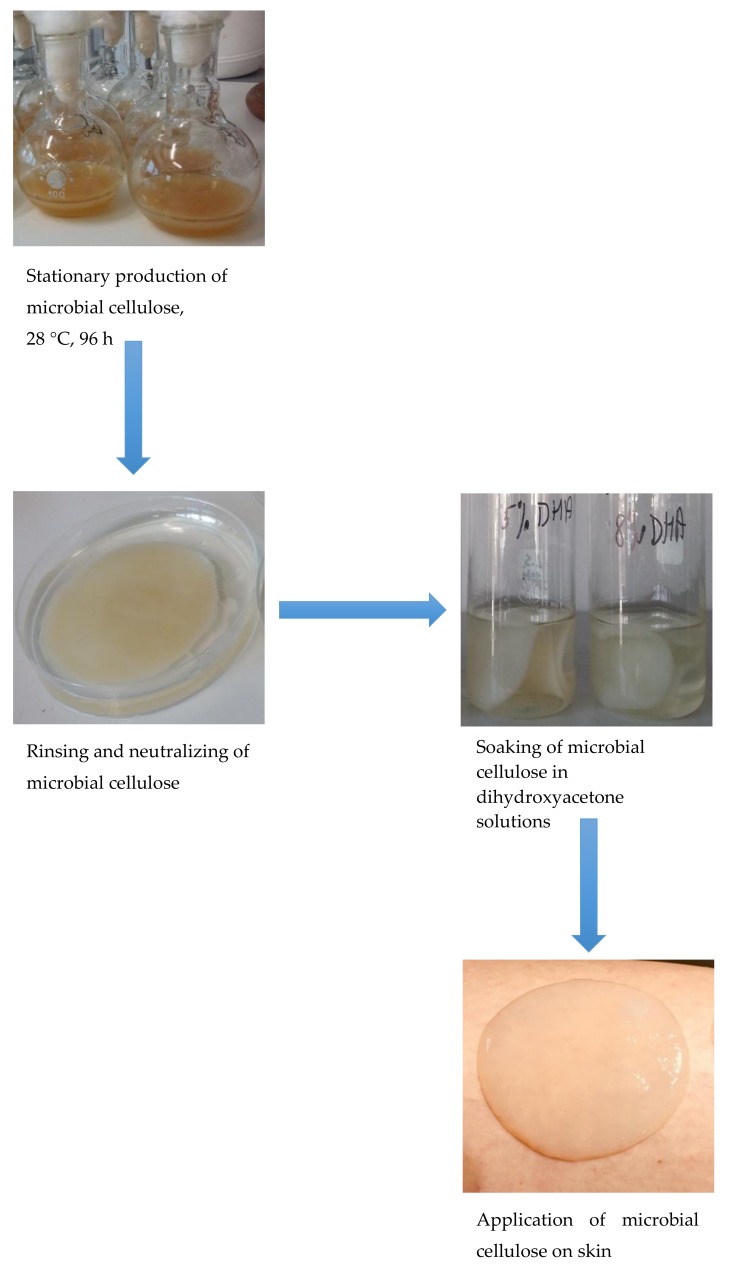
Process of preparing MC patches with DHA.

The processes of MC isolation, bacteria cells removal from MC, MC incubation with DHA, and MC patches with DHA application on skin were shown in Figure 3.

## 3. Results and Discussion

**Figure 4:**
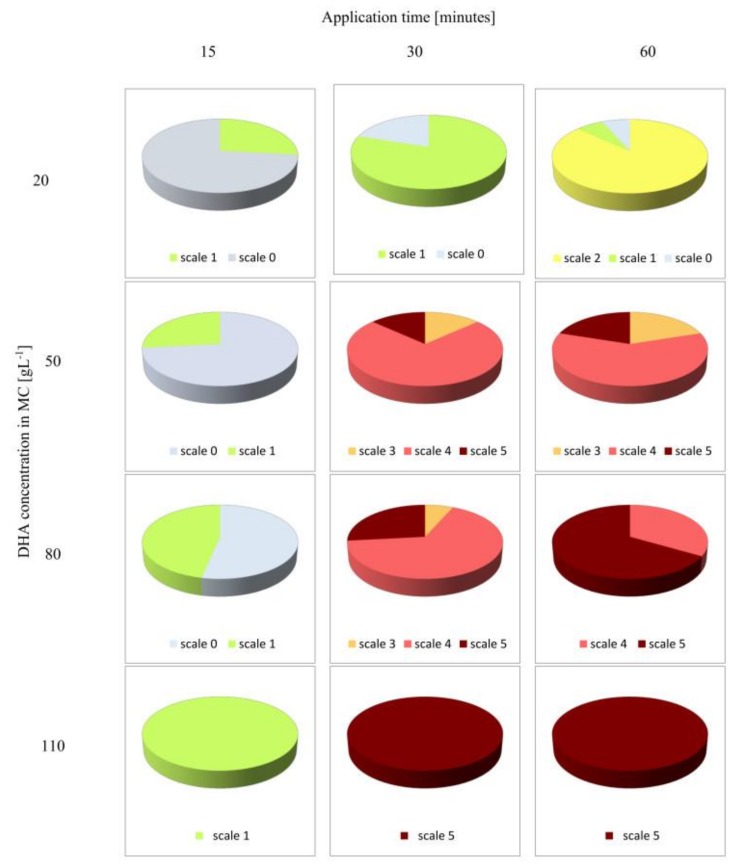
Results of the assessment of skin pigmentation changes conducted by 15 observers. Pigmentation intensity was rated from 0 to 5, where 0 means no color changes, and 5 means the darkest color compared to the control.

The average assessment of skin color darkening, after the application of MC patches with DHA made by 15 independent observers was presented in Figure 4.

The shade of brown color was dependent on DHA concentration in the incubation solution, i.e., it became darker with DHA concentration increase. 

The application of MC patches impregnated with 2% of DHA did not change the skin color after application for 15 min. After 30 min, a slight pigmentation of the skin was observed (scale 1). Extending the application time to 60 min resulted in a two-fold darkening of the skin compared to 30 min of patch application (Figure 4.)

**Figure 5:**
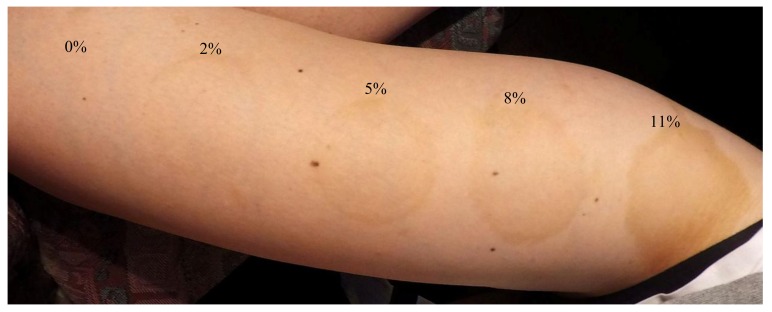
Skin coloring 12 h after removal of MC patches with DHA, which were applied for 30 min (concentration of DHA was expressed in %).

MC patches soaked in 5% of DHA did not change skin color after 15 min of application, although prolongation of the application time to 30 min resulted in pronounced pigmentation of the skin, evaluated at 4 on the five-point scale. The 60-min application did not change the color of the skin compared to the sample applied for 30 min (Figure 4). The effect of skin coloring after MC patches removal was shown in Figure 5.

In the case of applying MC patches soaked with DHA at the concentration of 80 (g·L^−1^), skin color changes were observed after 15 min of application. After 30 min, the skin color was rated at 4 in the five-point scale, and after the next 30 min at the maximum number of points in the scale (5).

**Figure 6:**
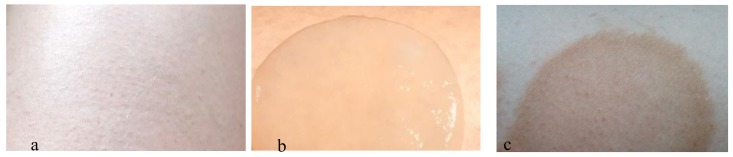
Skin area before (**a**), during (**b**) and after the application of MC with DHA (**c**) presents visible peripheral accentuation. Scale 1:2, 1 cm = 0.5 cm.

MC saturated with the highest applied concentration of DHA (110 g·L^−1^) after only 30 min of application caused the highest skin pigmentation, compared to that obtained after 60 min of its application (Figure 4). In the case of skin color change after MC application with higher concentrations of DHA (8% and 11%), peripheral accentuation was observed (Figure 6).

The experiment showed that duration of MC application had a greater influence on skin color changes than DHA concentration used to prepare the solution (in which the MC patches were incubated). Skin color was assessed equally (at 4 in the five-point scale) for MC patches incubated in both 5% (for 30 and 60 min) and 8% (for 30 min) DHA solution. The observers involved in the assessment of skin color found that the score of 4 in the scale corresponded to the most beautiful, acceptable and wanted effect that fitted best to the effect of natural tan. In addition, it had been noted that skin coloring with the use of MC containing DHA did not leave a characteristic unpleasant odor on skin typical of commercial self-tanning cosmetics. The obtained coloring effects lasted on the skin for 7 to 14 days. Some disadvantage for cellulose-based DHA application was the peripheral accentuation which was observed after using MC patches soaked with higher concentrations of DHA (Figure 6).

## 4. Conclusions

The results obtained in this study clearly indicate the feasibility of using MC as a biocarrier of DHA, which allows for contact with the stratum corneum, causing skin pigmentation. Application of MC patches with DHA in the concentration of 50 (g·L^−1^) for 30 min ensured the color of the skin, which was considered the closest to the natural wanted tan effect.

The described experiment was aimed at evaluating the possibility of using MC as a biocarrier of DHA, which would help in masking the effects of vitiligo. MC is a biomaterial that can be formed in any shapes. It is possible to design patches of MC which can match exactly to lesions where the color of skin has changed as a result of vitiligo. The MC structure allows accurate adhesion regardless of the shape and surface on which it is applied. 

Using DHA in the form of MC patches can be an alternative for patients with vitiligo because there is a real chance that this biomaterial will not cause skin allergies. Moreover, DHA applied onto the skin through microbial cellulose does not leave the specific, unpleasant odor, typical of commercial cosmetics containing DHA. It seems that this method can also be a cost-effective process compared with cosmetics production. The proposed method requires refinement, but it affords the possibility of designing biocarriers that could in the future provide personalized masking effects of vitiligo or creating a new formula of a self-tanning mask, which can be enriched with anti-aging compounds. The presented study has a scientific value. It showed that possibilities of microbial cellulose application can still be expanded.
